# Comparing the plant diversity between artificial forest and nature growth forest in a giant panda habitat

**DOI:** 10.1038/s41598-017-03895-3

**Published:** 2017-06-15

**Authors:** Dongwei Kang, Xiaorong Wang, Shuang Li, Junqing Li

**Affiliations:** 1 College of Forestry, Beijing Forestry University, Beijing, 100083 China; 2 Wanglang National Nature Reserve Administration Bureau, Mianyang, 622550 China

## Abstract

Artificial restoration is an important way to restore forests, but little is known about its effect on the habitat restoration of the giant panda. In the present study, we investigated the characteristics of artificial forest in the Wanglang Nature Reserve to determine whether through succession it has formed a suitable habitat for the giant panda. We compared artificial forest characteristics with those of natural habitat used by the giant panda. We found that the dominant tree species in artificial forest differed from those in the natural habitat. The artificial forest had lower plant species richness and diversity in the tree and shrub layers than did the latter, and its community structure was characterized by smaller tree and bamboo sizes, and fewer and lower bamboo clumps, but more trees and larger shrub sizes. The typical community collocation of artificial forest was a “*Picea asperata* + no-bamboo” model, which differs starkly from the giant panda’s natural habitat. After several years of restoration, the artificial forest has failed to become a suitable habitat for the giant panda. Therefore, a simple way of planting individual trees cannot restore giant panda habitat; instead, habitat restoration should be based on the habitat requirements of the giant panda.

## Introduction

Giant panda (*Ailuropoda melanoleuca*) is one of the flagship species in nature conservation. After years of protection efforts, its number and habitat area have reached 1864 individuals and 2,580,000 ha^1^, respectively. In the latest Red List of China’s Vertebrates, the giant panda is listed as a vulnerable species^2^. Recently, the IUCN also changed the status of this species from “endangered” to “vulnerable”^3^.

However, the current situation facing the giant panda remains very difficult, in that habitat fragmentation is still the main factor threatening the sustainable survival of its populations. For example, the total giant panda population is divided into 33 local populations, of which 24 are at higher risk of extinction^1^. Furthermore, human disturbances in and around giant panda habitat have been frequent^4, 5^, such as grazing^6^, road-building^7^, logging^8^, tourism^9^, hydropower stations^5^, etc, which leads to habitat degradation and destruction that affect the habitat use of the giant panda. To save this species, an urgent action needed is to restore degraded habitats and connect the fragmented habitats patches^10^.

Nevertheless, how to restore the giant panda habitat quicker and better is a challenging goal in research aimed at giant panda protection. Previous studies have shown that natural recovery is an effective way to achieve habitat recovery, but this process usually requires a relatively long time. For example, after approximately 50 years of natural recovery, logged habitats were able to function again as suitable giant panda habitat in the Wolong Nature Reserve^11^. Artificial restoration can rapidly increase the vegetation coverage^12^, but some studies have suggested that giant panda seldom and even hardly use the artificial forest^13–15^. Under this situation, only by identifying the defects of artificial restoration and rectifying them can we hope to better understand the reason for its failures, and thus improve its protective effect for the giant panda.

In this context, empirically evaluating the effect of artificial restoration could help summarise the experiences and lessons gained^12^, but there is little published literature on giant panda habitat restoration. In this study, we sought to evaluate the effect of artificial restoration as based on the characteristics of natural habitat used by the giant panda. We focused on the artificial forest that is currently distributed in the Wanglang Nature Reserve, one of the earliest nature reserves established for protecting giant panda^16^. Our main objective was to investigate the characteristics of the artificial forest to determine whether through succession it has successfully approximated suitable giant panda habitat. We were more interested in the tree and bamboo vegetation of the forest because they are the key components of giant panda habitat^17^, and thus, they affect the habitat selection of the giant panda^18^. We hope this timely field study could provide an important reference tool for practices to restore habitat of the giant panda and its co-occurring wild animals.

## Results

### Species composition

32 woody plant species (belonging to 25 genera and 15 families) were recorded in the artificial forest plots, and 32 woody plant species (belonging to 24 genera and 15 families) were recorded in the giant panda habitat plots.

14 tree species (belonging to 11 genera and 10 families) were recorded in the artificial forest plots, whereas 23 tree species (belonging to 19 genera and 13 families) were recorded in the giant panda habitat plots. 8 tree species were common to these two types of plots, for which the similarity coefficient was 0.43. The dominant tree species in the artificial forest plots was *Picea asperata*, which differed from the dominant ones in the giant panda habitat plots (Table 1).

**Table 1:** Dominant species of different layers in artificial forest plots and giant panda habitat plots.

Category	Artificial forest plots	Giant panda habitat plots
Tree layer	*Picea asperata*	*Abies faxoniana*, *Betula albosinensis*, *Betula utilis*, *Sabina saltuaria*, *Picea purpurea*
Shrub layer	*Picea asperata*, *Salix wallichiana*, *Philadelphus incanus*	*Philadelphus incanus*, *Ribes glaciale*, *Betula albosinensis*, *Lonicera microphylla*, *Abies faxoniana*, *Sorbus koehneana*, *Maddenia hypoleuca*
Regeneration layer	*Lonicera microphylla*, *Sorbaria arborea*, *Ribes glaciale*, *Ribes alpestre*, *Spiraea alpina*, *Daphne odora*, *Berberis sichuanica*, *Philadelphus incanus*	*Ribes glaciale*, *Abies faxoniana*, *Lonicera microphylla*, *Berberis sichuanica*

24 shrub species (belonging to 20 genera and 10 families) were recorded in the artificial forest plots, and 28 shrub species (belonging to 21 genera and 14 families) were recorded in the giant panda habitat plots. 14 shrub species were common to these two types of plots, for which the similarity coefficient was 0.54. *Philadelphus incanus* was the dominant shrub species in both the artificial forest plots and giant panda habitat plots (Table 1).

19 regeneration species (belonging to 16 genera and 11 families) were recorded in the artificial forest plots, and 16 regeneration species (belonging to 15 genera and 10 families) were recorded in giant panda habitat plots. 10 regeneration species were common to these two types of plots, for which the similarity coefficient was 0.57. *Lonicera microphylla*, *Ribes glaciale*, and *Berberis sichuanica* were the dominant regeneration species in both the artificial forest plots and giant panda habitat plots (Table 1).

Furthermore, the same bamboo species (*Fargesia denudata*) was recorded in artificial forest plots and giant panda habitat plots.

### Species richness and diversity

The species richness and Shannon-Wiener indexes of tree and shrub in artificial forest plots were all significantly lower than those of the giant panda habitat plots (all P-values < 0.05, Table 2). However, for the regeneration species, these two variables were not significantly different (P-values > 0.05, Table 2).

**Table 2:** Species richness and diversity index of artificial forest plot and giant panda habitat plot.

Variables	Mean (SD)		F or Z value	P value
	Artificial forest plot	Giant panda habitat plot		
Tree species richness	2.9(1.8)	5.4(1.8)	11.81	0.00
Shannon-Wiener index of tree	0.40(0.30)	1.40(0.46)	40.93	0.00
Shrub species richness	5.6(3.8)	9.6(3.2)	7.83	0.01
Shannon-Wiener index of shrub	1.10(0.83)	1.81(0.32)	−2.31	0.02
Regeneration species richness	4.3(3.7)	3.4(2.9)	0.46	0.50
Shannon-Wiener index of Regeneration	1.16(0.72)	0.87(0.53)	1.07	0.32

### Community structure

6 of the 10 variables differed significantly between artificial forest plots and giant panda habitat plots: tree number, tree size, shrub size, bamboo clump number, bamboo clump height, and bamboo size (all P-values < 0.05, Table 3). As compared to the giant panda habitat, the artificial forest was characterized by smaller tree size and bamboo size, fewer and lower bamboo clumps, but more trees and a larger shrub size (Table 3).

**Table 3:** Community structure of artificial forest plot and giant panda habitat plot.

Variables	Mean (SD)		F or Z value	P value
	Artificial forest plot	Giant panda habitat plot		
Tree number	46.6(26.0)	18.4(8.8)	−2.51	0.01
Tree size	17.8(5.0)	25.4(8.0)	7.81	0.01
Shrub number	34.1(29.2)	56.0(35.1)	−1.53	0.13
Shrub size	5.6(1.9)	3.8(0.9)	9.09	0.01
Regeneration number	23.3(23.0)	15.0(18.1)	−0.78	0.43
Regeneration height	69.2(24.8)	57.5(19.9)	1.35	0.26
Regeneration size	6.4(2.5)	6.6(3.1)	0.02	0.88
Bamboo clump number	5.7(13.4)	107.1(39.2)	−4.21	0.00
Bamboo clump height	107.0(18.9)	245.6(46.1)	−2.60	0.01
Bamboo size	5.0(1.3)	8.7(1.7)	−2.60	0.01

### Community collocation

In the artificial forest plots, *Picea asperata* was the important constructive species, as it appeared in 91.7% (11 of 12) of the artificial forest plots, whereas in the giant panda habitat plots, *Abies faxoniana* and *Betula albosinensis* were the important constructive species, as they appeared in 66.7% (8 of 12) and 41.7% (5 of 12) of these plots, respectively (Table 4). Meanwhile, 75.0% (9 of 12) of artificial forest plots had no bamboo in them, whereas every giant panda habitat plot sampled showed the presence of bamboo (Table 4).

**Table 4:** Community collocation model of artificial forest plot and giant panda habitat plot.

Artificial forest plot			Giant panda habitat plot		
Number	Constructive species	Bamboo	Number	Constructive species	Bamboo
1	*Picea asperata*	Absence	1	*Abies faxoniana*	Presence
2	*Picea asperata*	Absence	2	*Abies faxoniana*	Presence
3	*Picea asperata*	Absence	3	*Abies faxoniana*	Presence
4	*Picea asperata*	Absence	4	*Abies faxoniana*	Presence
5	*Picea asperata*	Absence	5	*Abies faxoniana*	Presence
6	*Picea asperata*	Absence	6	*Abies faxoniana*, *Betula albosinensis*	Presence
7	*Picea asperata*	Absence	7	*Abies faxoniana*, *Betula albosinensis*	Presence
8	*Picea asperata*	Absence	8	*Sabina saltuaria*, *Abies faxoniana*	Presence
9	*Picea asperata*	Absence	9	*Betula albosinensis*	Presence
10	*Picea asperata*	Presence	10	*Betula albosinensis*	Presence
11	*Picea asperata*	Presence	11	*Betula albosinensis, Acer longipes*	Presence
12	*Spiraea alpina*	Presence	12	*Picea purpurea*	Presence

## Discussion

Many factors can potentially affect habitat selection by animals^19^, such as food availability, water resources, quality shelter, places to hide, etc. Previously, we have found that the giant panda did not use the artificial forest habitat in the Wanglang Nature Reserve^15^. In this study, we still did not find the entity of giant panda and any trace of giant panda activity in the artificial forests and artificial forest plots, but we found a significant difference between the artificial forest and giant panda habitat in terms of plant species composition, community structure, and community collocation, which together may explain the giant panda’s lack of use of artificial forest in this area.

From the perspective of species composition, artificial forest had moderate similarity with the giant panda habitat in different layers after several years of succession. However, as the main planted tree species in Wanglang, *Picea asperata* was still the dominant species in the tree and shrub layers of artificial forest, which was different from the giant panda habitat, indicating that no substantial change has yet occurred in the tree and shrub layers of artificial forest. Furthermore, the richness and diversity of tree and shrub in artificial forest were all lower than those of giant panda habitat, suggesting that the species composition of artificial forest is simpler, which may have been driven by the planting of a single tree species. Thus, in spite of many years of succession, from this perspective, artificial forest is still very different from giant panda habitat. In addition, it is worth noting that the richness and diversity of regeneration in artificial forest were all had no significant differences with those of natural giant panda habitat, which may indicate that the artificial forest could on its way to restore naturally to the natural forest after a relatively longer time.

From the perspective of community structure, artificial forest differed significantly from giant panda habitat, except in the regeneration layer. Planting individual trees at a high density is the typical approach to afforestation; thus, it is no surprise that the tree number in artificial forest was more than twice that found in giant panda habitat. Trees with a larger size are more likely to provide suitable habitat for the giant panda; for example, both branches and under-the-tree environments are used as resting sites by the giant panda^16^. Although more trees were grown in the artificial forest, their size was significantly thinner than those trees growing in the giant panda habitat; to support pandas, so those thinner trees need further growth. Furthermore, the growth of shrubs in artificial forest was better than in the giant panda habitat plots, most likely because interspecific competition was weaker in the former where bamboo was small or absent. Bamboo is nearly the sole food resource of the giant panda, which reportedly can eat approximately 23–38 kg bamboo shoots per day^20^; therefore, an environment with sufficient bamboo is essential to qualify as giant panda habitat. However, in the present study, we found that bamboo was scarce in artificial forest, and even where it was present, its growth was very poor. Therefore, it is difficult to imagine how the giant panda can effectively use this kind of habitat, one that clearly cannot meet its food requirements.

**Figure 1:**
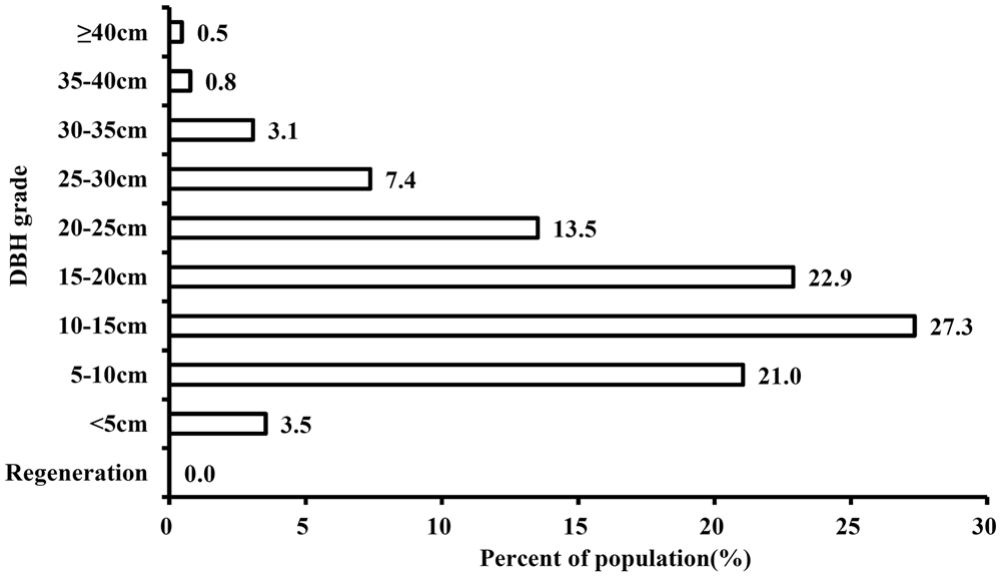
Population structure of *Picea asperata*.

“Tree-bamboo-giant panda” is an organic whole^17^, in that the typical giant panda habitat is usually described as consisting of tall trees and dense bamboo. It follows that tree and bamboo should be considered the important vegetation components of giant panda habitat. For the trees, the main constructive species of the artificial forest plots was *Picea asperata*, which differed completely from the constructive species of local giant panda habitat, such as *Abies faxoniana*, *Betula albosinensis*, *Picea purpurea*, etc. For the bamboo, 75.0% of artificial forest had no bamboo. For the three artificial forest plots that had bamboo, two of them were planted artificially and only one had regenerated naturally, and the number of bamboo clumps was only six. Presence of little or no bamboo does not characterise a suitable giant panda habitat. Thus, the current community collocation of artificial forest was mainly characterized by a “*Picea asperata* + no-bamboo” model (Table 4). Although the population structure of *Picea asperata* suggests that it is in decline, individuals with DBH at 5–20 cm in tree size still occupied >70% of the total population (Fig. 1). Hence, the situation of *Picea asperata* growing in artificial forests as the constructive species may not change in the short term. Furthermore, considering that the recovery of bamboo generally takes approximately 20–30 years^11^, it can be inferred that no major changes are likely to occur in artificial forest under natural conditions in the near future.

In this study, we found several striking differences between artificial forest and giant panda habitat in terms of their species composition, richness and diversity, community structure, and community collocation. Based on these results, we conclude that after several years of succession, the existing artificial forest in Wanglang does not provide a suitable habitat for the giant panda. Furthermore, according to our previous experience and the current status of artificial forest, we predict that no substantial changes would occur in the artificial forest in the near future. Consequently, a simple form of tree-planting cannot restore the giant panda habitat; instead, habitat restoration practices should be based on the habitat requirements of the giant panda.

## Methods

### Study area

The field work was carried out in the Wanglang National Nature Reserve, Pingwu County, Sichuan Province, China (103°50′–104°58′E, 32°49′–33°02′N). As an important giant panda habitat in the north Minshan Mountains^21^, Wanglang was established in 1963, covering an area of 32,297 hm^2^ ^4^ at an elevation ranging from 2,320 m to 4,891 m. The annual average precipitation at this nature reserve is approximately 862.5 mm, the lowest mean air temperature reached is −6.1 °C in January and the highest reached is 12.7 °C in July^22^. *Fargesia denudata* is the main bamboo species in this area.

### Field survey

To investigate the characteristics of artificial forest, we first selected 12 artificial forest stands in Wanglang, ten of them were about planted in 1960s, and the remaining two were planted in 1980s. Then, in each stand, one representative 20 m × 20 m plot was established. For the trees and shrubs in each plot, we measured and recorded their species name, the diameter at breast height (DBH), and the height of each individual/clump. For regeneration (woody plants below 1.3 m in height) in each plot we measured and recorded the species name, basal diameter, and height of each individual/clump. For the bamboos in each plot we measured and recorded their species name, the mean height of bamboo clump. Furthermore, to measure the bamboo size, we established five 1-m × 1-m bamboo quadrats distributed in the centre of each 20 m × 20 m plot and the centres of four 10 m × 10 m plots; in each bamboo quadrat, the basal diameter of five old bamboo individuals were measured randomly. This field survey was carried out in August 2015, and in July and August 2016.

To investigate the characteristics of natural giant panda habitat, we first selected 12 typical distribution areas of the giant panda in Wanglang Nature Reserve, according to previous studies^23^. In each area, one 20 m × 20 m plot that at least contained giant panda faeces was established to represent the giant panda habitat. The investigation method and time of sampling of these giant panda habitat plots were identical those used for the artificial forest plots.

**Figure 2:**
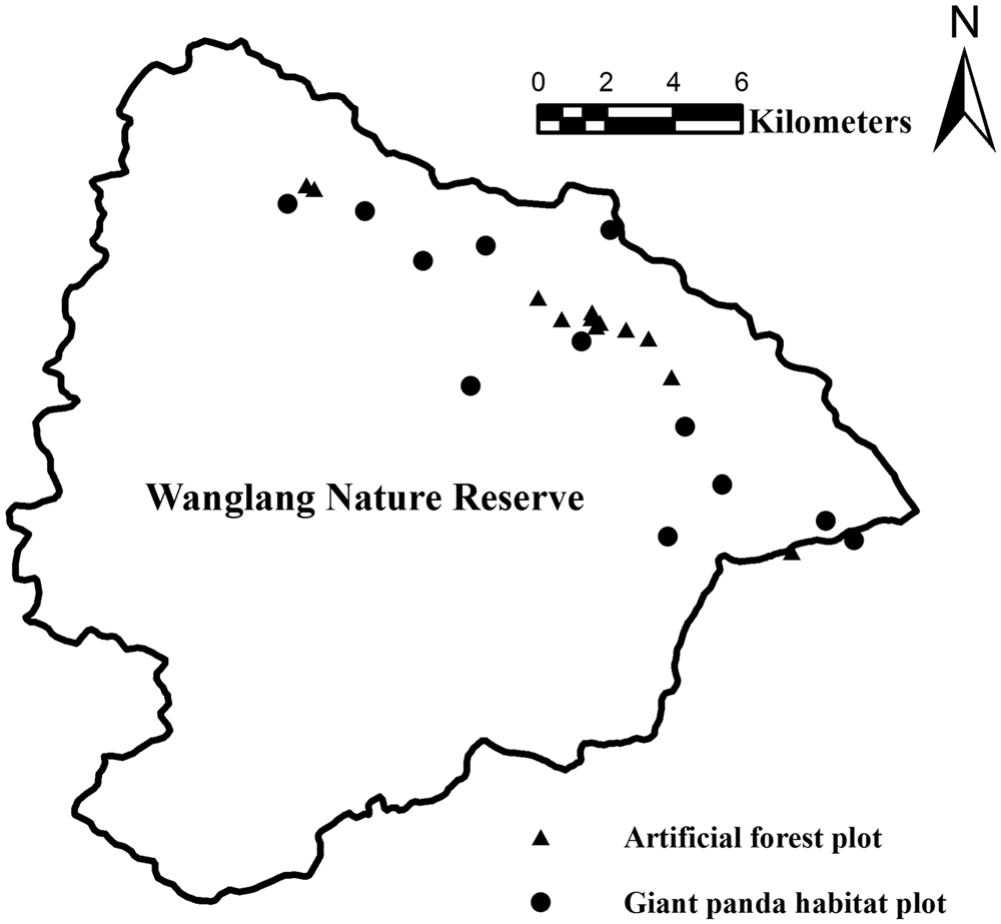
Distribution of plots in this study. This figure was generated by ArcGIS 10.2.

A total of 24 plots were sampled for analysis (12 artificial forest plots and 12 giant panda habitat plots) (Fig. 2).

### Data analysis

To describe the characteristics of the general plant species composition of artificial forest and giant panda habitat, we first counted the total number of family, genus, and species of woody plants and bamboo in all plots of artificial forest and giant panda habitat, respectively. Subsequently, we counted the species composition and calculated the similarity coefficient of different vegetation layers (tree, shrub and regeneration layers). Lastly, we compared the dominant species—those having an importance value exceeding 0.05—composition of different layers between the two types of plots.

To identify differences in plant species richness and diversity between the artificial forest and the giant panda habitat, we first counted the number of species and then calculated the Shannon-Wiener indexes of each artificial forest plot and giant panda habitat plot for the different layers. Then, for each layer, we compared the means of these two variables using the one-way ANOVA when statistical assumptions were met (the data were normally distributed, the variances were homogeneous) or Mann-Whitney *U* test when statistical assumptions were not met.

To detect the differences in plant community structure between the artificial forest and the giant panda habitat, we first defined and calculated 10 variables related to community structure according to the plot-level data (see Table 5). Subsequently, we used either the ANOVA or Mann-Whitney *U* test to separately compare the means of these variables between artificial forest plots and giant panda habitat plots.

**Table 5:** Definition of the variables used in this study.

Variable	Definition
Tree number	Total number of trees in 20 m × 20 m plot
Tree size (cm)	Average DBH of trees in 20 m × 20 m plot
Shrub number	Total number of shrubs in 20 m × 20 m plot
Shrub size (cm)	Average DBH of shrubs in 20 m × 20 m plot
Regeneration number	Total number of regenerations in 20 m × 20 m plot
Regeneration height (cm)	Average height of regenerations in 20 m × 20 m plot
Regeneration size (mm)	Average basal diameter of regenerations in 20 m × 20 m plot
Bamboo clump number	Total number of bamboo clumps in 20 m × 20 m plot
Bamboo clump height (cm)	Average height of bamboo clumps in 20 m × 20 m plot
Bamboo size (mm)	Average basal diameter of five 1 m × 1 m bamboo sites

To analyse the community collocation of artificial forest and giant panda habitat, we compared their constructive plant species from each plot; these were defined as species with a single or cumulative importance value exceeding 0.50 in the tree layer of a plot. We also examined the presence of bamboo in each artificial forest plot and giant panda habitat plot.

In this study, we used the Sorensen index as the similarity coefficient^24^:

1 $$SI=\frac{2c}{a+b}$$

where *a* and *b* represent the number of species in artificial forest plots and giant panda habitat plots, respectively, *c* stands for the number of species common to both artificial forest plots and giant panda habitat plots.

We used the Shannon-Wiener index as a measure of species diversity^24^:

2 $$H^{\prime} =-\sum {P}_{{\rm{i}}}\,\mathrm{ln}\,{P}_{{\rm{i}}}$$

where *P*_i_ stands for the proportion of the number of the *i* ^*th*^ species in each plot.

We used the relative basal area, the mean values of relative density and relative frequency, and the mean values of relative density and relative height to represent, respectively, the importance values of species in the tree layer, shrub layer, and regeneration layer.

The statistical significance level used in this study was set at 0.05.
